# Supplementary material for: Risk of major adverse cardiovascular events with aripiprazole versus olanzapine, quetiapine, and risperidone in severe mental illness: a target trial emulation
Source: Nat Commun. 2025 Dec 21;17:1083. doi: 10.1038/s41467-025-67843-w (PMC12852762; doi:10.1038/s41467-025-67843-w)
Supplement: Supplementary file 1 — Supplementary Information [file 41467_2025_67843_MOESM1_ESM.pdf]

Supplement to

"Risk of major adverse cardiovascular events with aripiprazole versus olanzapine, quetiapine, and risperidone in severe mental illness: a target trial emulation"

Alvin Richards-Belle, Naomi Launders, Sarah Hardoon, Kenneth K.C. Man, Neil M. Davies, Elvira Bramon, Joseph F. Hayes, David P.J. Osborn

CONTENTS

SUPPLEMENTARY METHODS 2
Power calculation.....2
Covariates.....2
SUPPLEMENTARY TABLES 3
Supplementary Table 1. Baseline characteristics of the unweighted sample. 3
Supplemental Table 2. Mean standardised differences between comparison groups before and after generalised overlap weighting. 5
Supplemental Table 3. MACE outcomes from the intention-to-treat analysis - absolute risks per 1,000 patients treated. 8
Supplementary Table 4. Adherence to the original treatment initiation strategy in the overlap weighted population.* 9
Supplemental Table 5. Five-year risk of MACE - intention-to-treat and per-protocol sensitivity analyses. 10
Supplementary Table 6. Comparison of the key design features for a hypothetical target trial and the target trial emulation comparing aripiprazole with olanzapine, quetiapine, and risperidone on the risk of major adverse cardiovascular events. 12
Supplementary Table 7. Baseline covariates included in estimation of generalised overlap weights. 14
Supplementary Table 8. Completeness of baseline covariates. 16
SUPLLEMENTARY FIGURES 17
Supplementary Figure 1. Standardised differences between comparison groups before and after generalised overlap weighting. 17
Supplementary Figure 2. Distribution of mean weights for each treatment strategy across imputed datasets. 18
Supplementary Figure 3. Forest plot of per-protocol sensitivity analyses for the five-year risk of MACE 19
Supplementary Figure 4. Confounding structure directed acyclic graph. 20
SUPPLEMENTARY REFERENCES 21

SUPPLEMENTARY METHODS

Power calculation

A formal power calculation did not guide the study as we planned to include all eligible patients. However, we conducted *a priori* illustrative power calculations for an unadjusted Cox regression analysis of the primary outcome (five-year MACE). Given the anticipated unequal group sizes and assuming an aripiprazole-comparator enrolment ratio of 2:5 (informed by previous research[1]), we estimated that detecting a statistically significant different hazard ratio of 1.10 ($p<0.05$) would require observing a total of 108 events for 80% power or 145 events for 90% power across each pairwise comparison (e.g., aripiprazole versus olanzapine combined).[2] Using an historical (1995-2004) cohort of patients initiating olanzapine, quetiapine, or risperidone with similar eligibility criteria as planned for the present study (n=14,479), the pooled comparator group probability of the primary outcome was estimated to be 7.08% (95% CI, 6.64% to 7.51%). This equates to a minimum required sample size in each pairwise comparison of 1,723 (95% CI, 1,625 to 1,837) for 80% power or 2,048 (95% CI, 1,931 to 2,184) for 90% power.

Covariates

Based on prior research and clinical expertise, we pre-specified the following baseline covariates as potential confounders of the treatment-outcome relationship: demographics (age, ethnicity, relative deprivation, sex), psychiatric history (SMI diagnosis, years from first SMI diagnosis to index date, prior use of non-study antipsychotics, prior psychiatric hospitalisation, olanzapine equivalent starting daily dose [computed using the Defined Daily Dose method][3]), comorbidities (alcohol misuse, angina, cardiac arrhythmia, diabetes, dyslipidaemia, hypertension, liver disease, renal failure, substance misuse), prescribed concomitant medications (anticoagulants, antidepressants, antidiabetics, antihypertensives, antiplatelets, anxiolytics, benzodiazepines, lipid-regulating medications, mood stabilisers, Z-drugs), body mass index category, index year, number of primary care consultations in the prior six months, prior physical health hospitalisation, and smoking status. Further details on how we operationalised and coded the variables is provided in Supplementary Table 7. Our assumed confounding structure is depicted using a directed acyclic graph in Supplementary Figure 4.

SUPPLEMENTARY TABLES

Supplementary Table 1. Baseline characteristics of the unweighted sample.

Characteristic	Aripiprazole N = 1,807	Olanzapine N = 7,965	Quetiapine N = 5,613	Risperidone N = 5,019
**Age (years), median (IQR)**	52 (45, 61)	53 (46, 65)	54 (46, 65)	56 (47, 69)
**Sex, n (%)**				
Female	1,021 (56.5%)	4,314 (54.2%)	3,470 (61.8%)	2,841 (56.6%)
Male	786 (43.5%)	3,651 (45.8%)	2,143 (38.2%)	2,178 (43.4%)
**Ethnicity, n (%)**				
Asian	150 (8.4%)	358 (4.6%)	226 (4.1%)	347 (7.0%)
Black	165 (9.2%)	453 (5.8%)	159 (2.9%)	494 (10.0%)
Mixed/Other	57 (3.2%)	180 (2.3%)	112 (2.0%)	139 (2.8%)
White	1,412 (79.1%)	6,851 (87.4%)	5,034 (91.0%)	3,959 (80.2%)
Unknown	23	123	82	80
**SMI diagnosis, n (%)**				
Bipolar disorder	507 (28.1%)	3,348 (42.0%)	3,305 (58.9%)	1,261 (25.1%)
Other non-organic psychoses	672 (37.2%)	3,128 (39.3%)	1,643 (29.3%)	2,513 (50.1%)
Schizophrenia	628 (34.8%)	1,489 (18.7%)	665 (11.8%)	1,245 (24.8%)
**Age at SMI diagnosis, median (IQR)**	43 (34, 53)	45 (37, 56)	46 (38, 57)	47 (38, 60)
**SMI diagnosis to index date (years), median (IQR)**	6 (0, 17)	3 (0, 15)	4 (0, 14)	3 (0, 15)
**Index of Multiple Deprivation (quintile), n (%)[1]**				
1 (Least deprived)	202 (11.2%)	1,212 (15.2%)	942 (16.8%)	686 (13.7%)
2	258 (14.3%)	1,353 (17.0%)	1,070 (19.1%)	766 (15.3%)
3	346 (19.1%)	1,489 (18.7%)	1,087 (19.4%)	931 (18.5%)
4	453 (25.1%)	1,809 (22.7%)	1,125 (20.0%)	1,250 (24.9%)
5 (Most deprived)	548 (30.3%)	2,102 (26.4%)	1,389 (24.7%)	1,386 (27.6%)
**Index year, median (IQR)**	2011 (2008, 2013)	2009 (2006, 2011)	2010 (2007, 2012)	2009 (2007, 2012)
**Comorbidities, n (%)[2]**				
Alcohol misuse	232 (12.8%)	1,114 (14.0%)	842 (15.0%)	587 (11.7%)
Angina	60 (3.3%)	212 (2.7%)	233 (4.2%)	235 (4.7%)
Arrhythmia	77 (4.3%)	282 (3.5%)	236 (4.2%)	231 (4.6%)
Diabetes	352 (19.5%)	543 (6.8%)	727 (13.0%)	746 (14.9%)
Dyslipidaemia	336 (18.6%)	1,003 (12.6%)	958 (17.1%)	849 (16.9%)
Hypertension	548 (30.3%)	1,856 (23.3%)	1,673 (29.8%)	1,587 (31.6%)
Liver disease	79 (4.4%)	280 (3.5%)	242 (4.3%)	191 (3.8%)
Renal disease	184 (10.2%)	657 (8.2%)	650 (11.6%)	532 (10.6%)
Substance misuse	175 (9.7%)	750 (9.4%)	496 (8.8%)	324 (6.5%)
**Concomitant medications, n (%)[3]**				
Prior antipsychotic use	515 (28.5%)	1,695 (21.3%)	1,284 (22.9%)	1,257 (25.0%)
Anticoagulants	37 (2.0%)	165 (2.1%)	134 (2.4%)	119 (2.4%)
Antidepressants	951 (52.6%)	4,404 (55.3%)	3,891 (69.3%)	2,427 (48.4%)

Characteristic	Aripiprazole N = 1,807	Olanzapine N = 7,965	Quetiapine N = 5,613	Risperidone N = 5,019
Antidiabetics	260 (14.4%)	280 (3.5%)	498 (8.9%)	536 (10.7%)
Antihypertensives	577 (31.9%)	2,159 (27.1%)	1,965 (35.0%)	1,707 (34.0%)
Antiplatelets	200 (11.1%)	692 (8.7%)	668 (11.9%)	699 (13.9%)
Anxiolytics	84 (4.6%)	304 (3.8%)	370 (6.6%)	211 (4.2%)
Benzodiazepines	480 (26.6%)	2,588 (32.5%)	2,142 (38.2%)	1,314 (26.2%)
Lipid regulating medications	442 (24.5%)	1,072 (13.5%)	1,122 (20.0%)	1,035 (20.6%)
Mood stabilisers	443 (24.5%)	2,311 (29.0%)	2,252 (40.1%)	968 (19.3%)
Z drugs	337 (18.6%)	1,897 (23.8%)	1,631 (29.1%)	912 (18.2%)
**Primary care consultations in last 6m, median (IQR)**	6 (3, 10)	5 (2, 10)	7 (3, 11)	6 (3, 10)
**Smoking status, n (%)**				
Current smoker	784 (43.7%)	3,711 (47.5%)	2,492 (44.8%)	2,105 (42.5%)
Ex-smoker	294 (16.4%)	1,047 (13.4%)	865 (15.6%)	748 (15.1%)
Never smoked	718 (40.0%)	3,053 (39.1%)	2,203 (39.6%)	2,101 (42.4%)
Unknown	11	154	53	65
**BMI category, n (%)**				
<18.5	28 (2.1%)	196 (4.0%)	97 (2.5%)	87 (2.6%)
≥18.5 to <25	355 (26.6%)	1,902 (38.6%)	1,177 (30.4%)	1,108 (33.3%)
≥25 to <30	390 (29.3%)	1,673 (33.9%)	1,311 (33.9%)	1,080 (32.4%)
≥30	560 (42.0%)	1,158 (23.5%)	1,283 (33.2%)	1,056 (31.7%)
Unknown	474	3,036	1,745	1,688
**BMI (kg/m2), mean (SD)**	29.5 (7.0)	26.6 (5.6)	28.2 (6.3)	27.8 (6.2)
Unknown	477	3,051	1,768	1,697
**Body weight (kg), median (IQR)**	80 (67, 95)	73 (63, 85)	77 (65, 90)	75 (64, 88)
Unknown	468	3,022	1,741	1,668
**Psychiatric hospitalisation in prior 2y, n (%)**	555 (30.7%)	2,818 (35.4%)	1,437 (25.6%)	1,355 (27.0%)
**Physical health hospitalisation in prior 2y, n (%)**	560 (31.0%)	2,491 (31.3%)	1,768 (31.5%)	1,583 (31.5%)
**Starting daily dose (Olanzapine equivalent) (mg), mean (SD)[4]**	7.3 (4.0)	8.6 (5.7)	4.3 (4.5)	4.3 (3.3)
Unknown	342	1,405	1,373	1,047

SMI, severe mental illness; BMI, body mass index; mg, milligrams; kg, kilograms; SD, standard deviation; IQR, interquartile range.

Characteristics are reported prior to imputation.

[1] Quintile of the 2019 English Index of Multiple Deprivation - a small-area measure of relative deprivation, defined according to the patient's residential postcode or, where this was missing (unweighted n=42), the primary care practice postcode.

[2] Comorbidities determined at point in the patient's medical history up to and including the index date.

[3] Concomitant medications defined according to prescriptions on, or in the two years prior to, the index date (prior antipsychotic use considered only prescriptions prior).

[4] Calculated according to the Defined Daily Dose method[3] and expressed as an olanzapine equivalent dose.

Supplemental Table 2. Mean standardised differences between comparison groups before and after generalised overlap weighting.

	Olanzapine vs. Aripiprazole		Quetiapine vs. Aripiprazole		Risperidone vs. Aripiprazole	
**Variable**	Unweighted	Overlap weighted	Unweighted	Overlap weighted	Unweighted	Overlap weighted
Age	0.14	0.02	0.20	0.02	0.35	0.01
Age (squared)	0.14	0.02	0.21	0.03	0.36	0.01
Sex	0.05	0.01	0.11	0.01	0.00	0.03
Ethnicity - Asian	0.15	0.00	0.17	0.00	0.05	0.02
Ethnicity - Black	0.13	0.01	0.25	0.02	0.03	0.01
Ethnicity - Mixed/Other	0.05	0.01	0.07	0.02	0.02	0.00
Ethnicity - White	0.22	0.00	0.32	0.01	0.03	0.02
SMI diagnosis - Bipolar disorder	0.29	0.01	0.64	0.00	0.06	0.01
SMI diagnosis - Other non-organic psychoses	0.04	0.02	0.16	0.03	0.27	0.01
SMI diagnosis - Schizophrenia	0.36	0.02	0.52	0.04	0.23	0.01
Time from diagnosis to index	0.09	0.01	0.14	0.01	0.09	0.01
Time from diagnosis to index (squared)	0.04	0.01	0.10	0.01	0.02	0.00
Deprivation quintile - 1 (Least deprived)	0.12	0.01	0.17	0.02	0.07	0.01
Deprivation quintile - 2	0.07	0.01	0.13	0.01	0.03	0.01
Deprivation quintile - 3	0.01	0.00	0.01	0.01	0.02	0.03
Deprivation quintile - 4	0.06	0.01	0.12	0.02	0.00	0.01
Deprivation quintile - 5 (Most deprived)	0.09	0.03	0.12	0.01	0.06	0.02
Index year	0.51	0.04	0.24	0.07	0.42	0.08
Index year (squared)	0.51	0.04	0.24	0.07	0.42	0.08
**Comorbidities**						
Alcohol misuse	0.03	0.00	0.06	0.01	0.03	0.02
Angina	0.04	0.01	0.05	0.00	0.08	0.01
Arrhythmia	0.04	0.02	0.00	0.01	0.02	0.01
Diabetes	0.36	0.00	0.19	0.02	0.13	0.01
Dyslipidaemia	0.16	0.00	0.04	0.03	0.05	0.01

	Olanzapine vs. Aripiprazole		Quetiapine vs. Aripiprazole		Risperidone vs. Aripiprazole	
**Variable**	Unweighted	Overlap weighted	Unweighted	Overlap weighted	Unweighted	Overlap weighted
Hypertension	0.16	0.01	0.01	0.01	0.03	0.00
Liver disease	0.04	0.01	0.00	0.00	0.03	0.01
Renal disease	0.07	0.04	0.05	0.01	0.01	0.01
Substance misuse	0.01	0.01	0.03	0.01	0.11	0.01
**Concomitant medications**						
Prior antipsychotic use	0.16	0.00	0.13	0.01	0.08	0.03
Anticoagulants	0.00	0.01	0.02	0.01	0.02	0.01
Antidepressants	0.05	0.02	0.34	0.02	0.09	0.02
Antidiabetics	0.36	0.00	0.18	0.03	0.12	0.01
Antihypertensives	0.10	0.01	0.07	0.01	0.05	0.01
Antiplatelets	0.08	0.01	0.03	0.01	0.09	0.01
Anxiolytics	0.04	0.03	0.09	0.01	0.02	0.03
Benzodiazepines	0.13	0.02	0.25	0.02	0.01	0.02
Lipid-regulating medications	0.27	0.01	0.11	0.01	0.10	0.01
Mood stabilisers	0.10	0.02	0.35	0.01	0.12	0.00
Z-drugs	0.13	0.02	0.25	0.01	0.01	0.02
Number of primary care consults	0.07	0.00	0.11	0.02	0.04	0.01
Number of primary care consults (squared)	0.06	0.01	0.06	0.00	0.04	0.01
Smoking status - Current smoker	0.08	0.01	0.02	0.01	0.02	0.01
Smoking status - Ex-smoker	0.08	0.02	0.02	0.01	0.04	0.03
Smoking status - Never smoked	0.02	0.01	0.01	0.01	0.05	0.01
BMI category - <18.5	0.06	0.01	0.03	0.03	0.02	0.01
BMI category - ≥18.5 to <25	0.23	0.00	0.09	0.01	0.16	0.02
BMI category - ≥25 to <30	0.07	0.01	0.07	0.01	0.05	0.02
BMI category - ≥30	0.32	0.01	0.17	0.03	0.20	0.04
Psychiatric hospitalisation	0.10	0.04	0.11	0.01	0.08	0.01

Variable	Olanzapine vs. Aripiprazole		Quetiapine vs. Aripiprazole		Risperidone vs. Aripiprazole	
	Unweighted	Overlap weighted	Unweighted	Overlap weighted	Unweighted	Overlap weighted
Physical health hospitalisation	0.01	0.00	0.01	0.03	0.01	0.00
Starting daily dose	0.31	0.05	0.73	0.06	0.73	0.03
Starting daily dose (squared)	0.40	0.04	0.39	0.06	0.47	0.02
Starting daily dose (cubed)	0.33	0.02	0.17	0.06	0.23	0.02

SMI, severe mental illness; BMI, body mass index. Calculated as the average across all 25 imputed datasets.

Supplemental Table 3. MACE outcomes from the intention-to-treat analysis - absolute risks per 1,000 patients treated.

	Aripiprazole	Olanzapine	Quetiapine	Risperidone
**Primary outcome**				
MACE, 5y	50.6 (37.7, 56.6)	49.4 (40.3, 53.7)	50.0 (40.3, 58.5)	57.6 (45.6, 62.6)
**Secondary outcomes**				
MACE, 6m	5.3 (2.2, 6.0)	8.1 (4.7, 8.3)	8.8 (4.6, 9.6)	7.5 (4.1, 7.7)
Non-fatal stroke, 5y	14.2 (8.1, 17.4)	11.4 (8.1, 13.6)	13.6 (9.4, 17.0)	14.3 (10.0, 16.4)
Non-fatal myocardial infarction, 5y	7.1 (3.1, 9.7)	10.1 (7.2, 11.8)	7.0 (4.2, 9.7)	9.4 (6.1, 11.7)
Cardiovascular death, 5y	30.8 (21.7, 36.8)	31.4 (24.8, 35.1)	32.6 (25.2, 40.2)	37.1 (28.3, 41.8)

Values are absolute risks with 95% confidence intervals. MACE, major adverse cardiovascular events.

Risks were predicted using a weighted pooled logistic regression model. Multiple imputation was used to handle missing covariate data, with 25 imputed datasets generated. Point estimates were pooled across imputed datasets according to Rubin's rules. We derived bias-corrected and accelerated percentile-based 95% confidence intervals from 12,500 estimates obtained by bootstrapping the procedure for estimating weights and outcome models, using 500 samples (stratified by treatment) per imputed dataset.

Supplementary Table 4. Adherence to the original treatment initiation strategy in the overlap weighted population.*

Characteristic	Aripiprazole N = 1,436	Olanzapine N = 4,523	Quetiapine N = 2,244	Risperidone N = 2,904
Remained adherent	785 (54.7%)	2,526 (55.8%)	1,363 (60.7%)	1,682 (57.9%)
Became non-adherent	651 (45.3%)	1996 (44.1%)	881 (39.3%)	1,222 (42.1%)
**Non-adherence reason**				
Discontinued	337 (51.8%)	1,167 (58.5%)	426 (48.4%)	688 (56.3%)
Switched	314 (48.2%)	829 (41.5%)	455 (51.6%)	534 (43.7%)
Time to non-adherence (months)	6 (4, 19)	9 (6, 23)	10 (6, 23)	6 (6, 21)

** Proportions calculated using weighting applied to one imputed dataset. Adherence was determined based on prescription records during follow-up time in which patients remained registered at primary care practices contributing data to CPRD; a similar proportion across groups ended registration during the study period (unweighted, range 16 to 17%) or were administratively censored (2 to 3%).*

Supplemental Table 5. Five-year risk of MACE - intention-to-treat and per-protocol sensitivity analyses.

	Ratio (95% CI)	
**Sensitivity analysis**	Intention-to-treat	Per-protocol
**Overlap weighting (PLR) [Primary]**		
Aripiprazole vs. Olanzapine	1.03 (0.77, 1.30)	0.77 (0.51, 1.10)
Aripiprazole vs. Quetiapine	1.02 (0.71, 1.30)	0.90 (0.53, 1.24)
Aripiprazole vs. Risperidone	0.88 (0.66, 1.15)	0.58 (0.39, 0.84)
**Overlap weighting (Cox)**		
Aripiprazole vs. Olanzapine	1.01 (0.76, 1.34)	0.73 (0.48, 1.12)
Aripiprazole vs. Quetiapine	0.92 (0.68, 1.26)	0.79 (0.49, 1.25)
Aripiprazole vs. Risperidone	0.89 (0.67, 1.17)	0.59 (0.39, 0.91)
**Overlap weighting (PLR), complete case**		
Aripiprazole vs. Olanzapine	0.84 (0.71, 1.28)	0.66 (0.45, 1.16)
Aripiprazole vs. Quetiapine	0.76 (0.63, 1.25)	0.84 (0.56, 1.51)
Aripiprazole vs. Risperidone	0.70 (0.60, 1.16)	0.48 (0.35, 0.87)
**Overlap weighting (PLR), more inclusive eligibility**		
Aripiprazole vs. Olanzapine	1.05 (0.80, 1.34)	0.89 (0.62, 1.27)
Aripiprazole vs. Quetiapine	0.87 (0.65, 1.15)	0.86 (0.58, 1.23)
Aripiprazole vs. Risperidone	0.87 (0.66, 1.12)	0.64 (0.44, 0.91)
**IP weighting (PLR)**		
Aripiprazole vs. Olanzapine	0.94 (0.72, 1.18)	0.71 (0.44, 1.04)
Aripiprazole vs. Quetiapine	0.79 (0.60, 1.01)	0.65 (0.40, 0.95)
Aripiprazole vs. Risperidone	0.75 (0.58, 0.95)	0.51 (0.33, 0.77)
**IP weighting (Cox)**		
Aripiprazole vs. Olanzapine	0.95 (0.70, 1.30)	0.68 (0.43, 1.07)
Aripiprazole vs. Quetiapine	0.79 (0.57, 1.09)	0.63 (0.38, 1.02)
Aripiprazole vs. Risperidone	0.76 (0.55, 1.02)	0.52 (0.33, 0.83)
**Covariate adjustment (Cox)**		
Aripiprazole vs. Olanzapine	0.97 (0.76, 1.24)	0.69 (0.47, 1.02)
Aripiprazole vs. Quetiapine	0.95 (0.73, 1.23)	0.68 (0.45, 1.02)
Aripiprazole vs. Risperidone	0.86 (0.66, 1.10)	0.63 (0.43, 0.93)
**Overlap weighting with IPCW (PLR), baseline[1]**		
Aripiprazole vs. Olanzapine	-	0.80 (0.54, 1.13)
Aripiprazole vs. Quetiapine	-	0.97 (0.58, 1.33)
Aripiprazole vs. Risperidone	-	0.60 (0.40, 0.87)
**Overlap weighting with IPCW (PLR), baseline + time-varying comorbidities[1]**		
Aripiprazole vs. Olanzapine	-	0.79 (0.52, 1.11)
Aripiprazole vs. Quetiapine	-	0.95 (0.57, 1.31)
Aripiprazole vs. Risperidone	-	0.59 (0.39, 0.86)

MACE, major adverse cardiovascular event; PLR, pooled logistic regression; IP, inverse probability; IPCW, inverse probability of censoring weighting; CI, confidence interval.

Estimates are risk or hazard ratios (as appropriate), with 95% confidence intervals (CIs). Risk ratios from the primary analysis (overlap weighting [PLR]) are shown for comparison. CIs for PLR results were derived from the distribution of 2,500 estimates

obtained via bootstrapping (i.e., 100 samples per imputed dataset). Bootstrap samples for the IP weighted PLR were increased to 500 due to instability at 100.

[1] *Censor weights (stabilised and trimmed at the 99.5th percentile) were estimated from the time-dependent probability of censoring and combined with the generalised overlap weights. In one model ("baseline"), censor weights were estimated based on pre-specified baseline covariates only; a second model ("baseline + time-varying comorbidities") additionally included a monthly time-varying cumulative count of comorbidities during follow-up.*

Supplementary Table 6. Comparison of the key design features for a hypothetical target trial and the target trial emulation comparing aripiprazole with olanzapine, quetiapine, and risperidone on the risk of major adverse cardiovascular events.

Trial design	Hypothetical target trial	Target trial emulation
**Inclusion criteria**	Clinical decision to initiate aripiprazole, olanzapine, quetiapine, or risperidone as oral monotherapy in primary care for the first time	• Incident prescription of study antipsychotic identified during study period (not explicitly labelled for *pro re nata* use)
		• No prescription for another antipsychotic on index date (or a long-acting injectable antipsychotic in prior 90d)
	Severe mental illness diagnosis	Severe mental illness diagnosis recorded in primary care (prior to, or up to 30d after, index date)
	Age 40 to 99	Aged 40 to 99 on index date
	-	Registered at primary care practice for at least 6m at index date
	-	Eligible for linkage to HES/ONS data
**Exclusion criteria**	Dementia	Dementia diagnosis recorded in primary care or HES on, or prior to, index date
	Previous stroke/myocardial infarction	Myocardial infarction/stroke diagnosis recorded in primary care or HES on, or prior to, index date
**Study period**	2005-2014	2005-2014
**Treatment strategies**	Intention-to-treat: Initiation of aripiprazole, olanzapine, quetiapine, or risperidone as oral monotherapy for the first time.<br><br>Per-protocol: Initiation and continued prescription of aripiprazole, olanzapine, quetiapine, or risperidone as oral monotherapy for the first time in primary care. Switch/discontinuation permitted in the presence of a recorded adverse reaction.	Intention-to-treat: Initiation of aripiprazole, olanzapine, quetiapine, or risperidone as oral monotherapy for the first time in primary care.<br><br>Per-protocol: Initiation and continued prescription of aripiprazole, olanzapine, quetiapine, or risperidone as oral monotherapy for the first time in primary care. Switch/discontinuation permitted in the presence of a recorded adverse reaction.
**Assignment procedure**	Randomisation	Patients assigned to the treatment strategy that their data is consistent with on the index date. Overlap weighting based on baseline confounders used in an attempt to emulate randomisation. Candidate confounders selected based on prior research and clinical expertise, guided by a directed acyclic graph.
**Primary outcome**	Major adverse cardiovascular event (MACE: composite of hospitalisation for non-fatal acute myocardial infarction or stroke and cardiovascular death) by 5y post-randomisation.	Major adverse cardiovascular event (MACE: composite of hospitalisation for non-fatal acute myocardial infarction or stroke and cardiovascular death) by 5y post-index date.
**Secondary outcomes**	• MACE by 6m.<br>• Non-fatal acute myocardial infarction by 5y.	• MACE by 6m.<br>• Non-fatal acute myocardial infarction by 5y.

	• Non-fatal stroke by 5y.<br>• Cardiovascular death by 5y.	• Non-fatal stroke by 5y.<br>• Cardiovascular death by 5y.
**Start of follow-up (time zero)**	Date of randomisation	The date on which eligibility criteria were met and the study antipsychotic was first prescribed.
**Follow-up duration (primary outcome)**	Intention-to-treat: Patients censored at earliest of first incident MACE, non-cardiovascular death, 5y post-randomisation, or administrative censoring.<br><br>Per-protocol: As above, with additional censoring at earliest of switch to another antipsychotic, addition of another antipsychotic to treatment, discontinuation (i.e., 6m following last prescription), de-registration from primary care, or practice last data collection date.	Intention-to-treat: Patients censored at earliest of first incident MACE, non-cardiovascular death, 5y post-index date, or administrative censoring.<br><br>Per-protocol: As above, with additional censoring at earliest of switch to another antipsychotic, addition of another antipsychotic to treatment, discontinuation (i.e., 6m following last prescription), de-registration from primary care, or practice last data collection date.
**Causal contrasts**	Intention-to-treat (primary) and per-protocol (supplementary)	Observational analogues of intention-to-treat (primary) and per-protocol (supplementary)
**Primary estimand**	Average treatment effect	Average treatment effect in the overlap weighted population
**Analysis plan**	Pooled logistic regression model to estimate outcome risks (differences and ratios) and hazard ratios of the aripiprazole initiation strategy versus comparator strategies.<br><br>Sensitivity analyses covering other approaches to modelling, confounding, missing data, and censoring.	Pooled logistic regression model to estimate outcome risks (differences and ratios) and hazard ratios of the aripiprazole initiation strategy versus comparator strategies.<br><br>Sensitivity analyses covering other approaches to modelling, confounding, missing data, and censoring.

** Table adapted from Hernan and Robins (2016)[4]*

HES, hospital episode statistics; ONS, office for national statistics; MACE, major adverse cardiovascular event.

Supplementary Table 7. Baseline covariates included in estimation of generalised overlap weights.

Variable	Timing	Type	Functional form	Categorical values	Data source
**Demographics**					
Age	At index year	Continuous	Linear, quadratic	-	CPRD
Ethnicity[1]	Most frequently recorded	Categorical	Linear	Asian, Black, Mixed/Other, White	CPRD & HES
Quintile of 2019 English Index of Multiple Deprivation	Most recent	Categorical	Linear	1 (Least deprived), 2, 3, 4, 5 (Most deprived)	CPRD
Sex	As recorded	Binary	Linear	Female, Male	CPRD
Antipsychotic dose (olanzapine equivalent)	At index date	Continuous	Linear, quadratic, cubic, interaction (x SMI diagnosis)	-	CPRD
Prior psychiatric hospitalisation	In prior 2y	Binary	Linear	-	HES
Prior use of antipsychotics	In prior 2y	Binary	Linear	-	CPRD
SMI diagnosis	Most recent at index date[2]	Categorical	Linear, Interaction (x antipsychotic dose)	Bipolar disorder, Other non-organic psychoses, Schizophrenia	CPRD
Years from first SMI diagnosis to index date	At index date	Continuous	Linear, quadratic	-	CPRD
**Comorbidities[3]**					
Alcohol misuse<br>Angina<br>Cardiac arrhythmia<br>Diabetes<br>Dyslipidaemia<br>Hypertension<br>Liver disease<br>Renal failure<br>Substance misuse	Ever-recorded prior to index date	Binary	Linear	-	CPRD & HES
**Prescribed concomitant medication[4]**					

Anticoagulants<br>Antidepressants<br>Antidiabetics<br>Antihypertensives<br>Antiplatelets<br>Anxiolytics<br>Benzodiazepines<br>Lipid-regulating medications<br>Mood stabilisers<br>Z-drugs	In prior 2y	Binary	Linear	-	CPRD
**Other**					
Body mass index category	Most recent at index date	Categorical	Linear	<18.5, ≥18.5 to <25, ≥25 to <30, ≥30	CPRD
Calendar year of index date	At index date	Continuous	Linear, quadratic	-	CPRD
Number of primary care consultations	In prior 6m	Continuous	Linear, quadratic	-	CPRD
Prior physical health hospitalisation	In prior 2y	Binary	Linear	-	HES
Smoking status	Most recent at index date	Categorical	Linear	Current smoker, Ex-smoker, Never smoked	CPRD

SMI, severe mental illness.

[1] Where a patient had multiple ethnicity categories recorded, the most frequently recorded was used, or the most recent, if frequencies were equal.

[2] SMI diagnosis could be recorded up to 30 days after the index date to allow for communication between health services and/or recording delays.

[3] Defined according to diagnostic codes recorded on or prior to the index date.

[4] Defined according to product codes, as recorded in prescription records, on or within the two years prior to the index date.

Supplementary Table 8. Completeness of baseline covariates.

Variable	Completeness (%)
Age	100
Alcohol misuse	100
Angina	100
Anticoagulant prescription	100
Antidepressant prescription	100
Antidiabetic prescription	100
Antihypertensive prescription	100
Antiplatelet prescription	100
Anxiolytic prescription	100
Arrhythmia	100
Benzodiazepine prescription	100
Diabetes	100
Dyslipidaemia	100
Hypertension	100
Index of Multiple Deprivation	100
Index year	100
Lipid regulating medication prescription	100
Liver disease	100
Mood stabiliser prescription	100
Primary care consultations in last 6m	100
Prior antipsychotic use	100
Prior physical health hospitalisation	100
Prior psychiatric hospitalisation	100
Renal disease	100
SMI diagnosis	100
Sex	100
Substance misuse	100
Years from SMI diagnosis to index date	100
Z-drug prescription	100
Smoking status	98.61
Ethnicity	98.49
Starting daily dose	79.58
BMI category	65.97

SMI, severe mental illness; BMI, body mass index.

SUPLLEMENTARY FIGURES

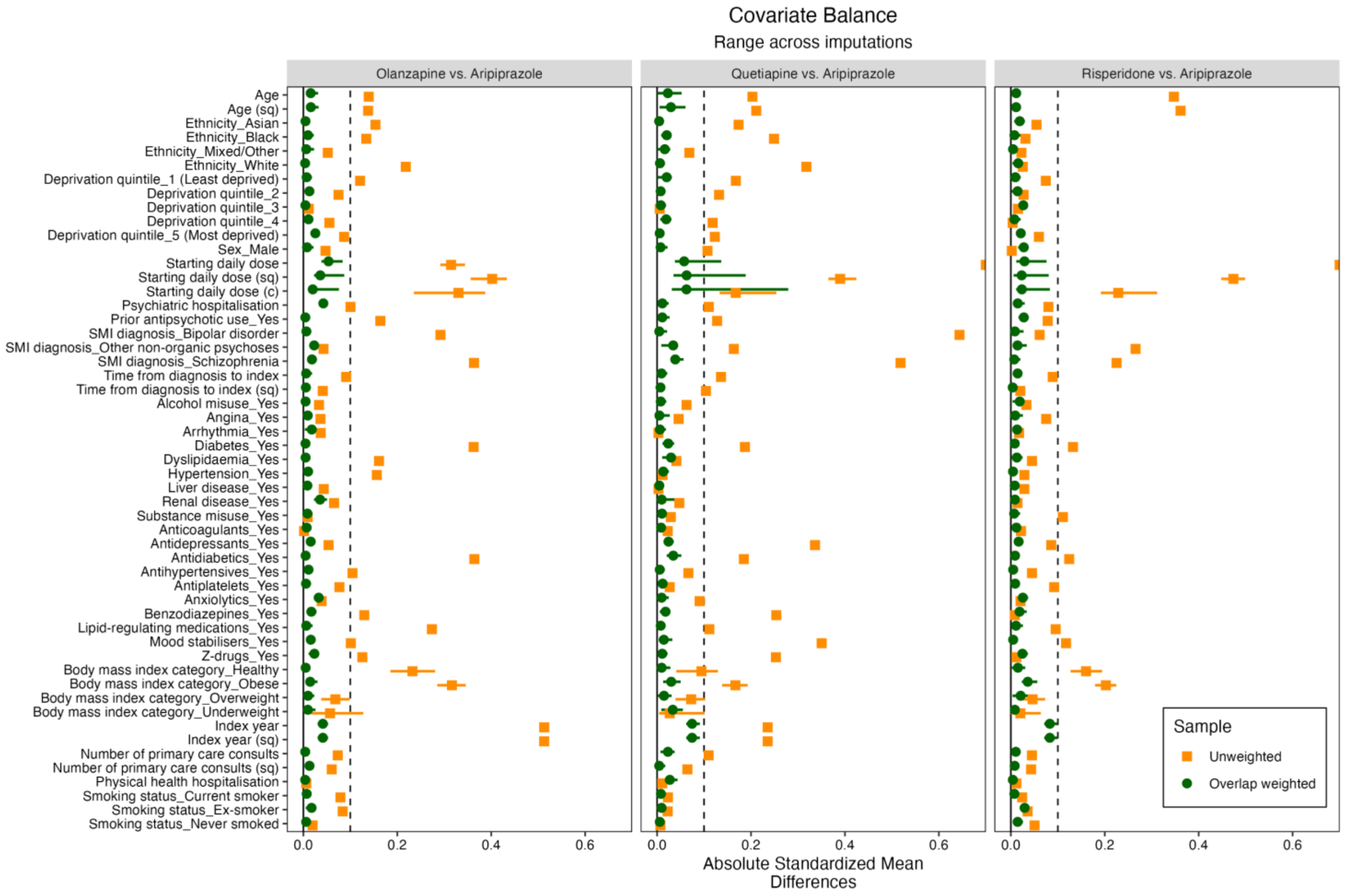

Supplementary Figure 1. Standardised differences between comparison groups before and after generalised overlap weighting.

Standardised mean differences in covariates in the unweighted sample and in the overlap weighted population between the aripiprazole initiation strategy and each of the comparator antipsychotic strategies (averaged over the 25 imputed datasets). All covariates demonstrated standardised differences less than 0.10 following weighting. Weighting and balance diagnostics performed using MatchThem and cobalt R packages.[5,6] Source data are provided as a Source Data file.

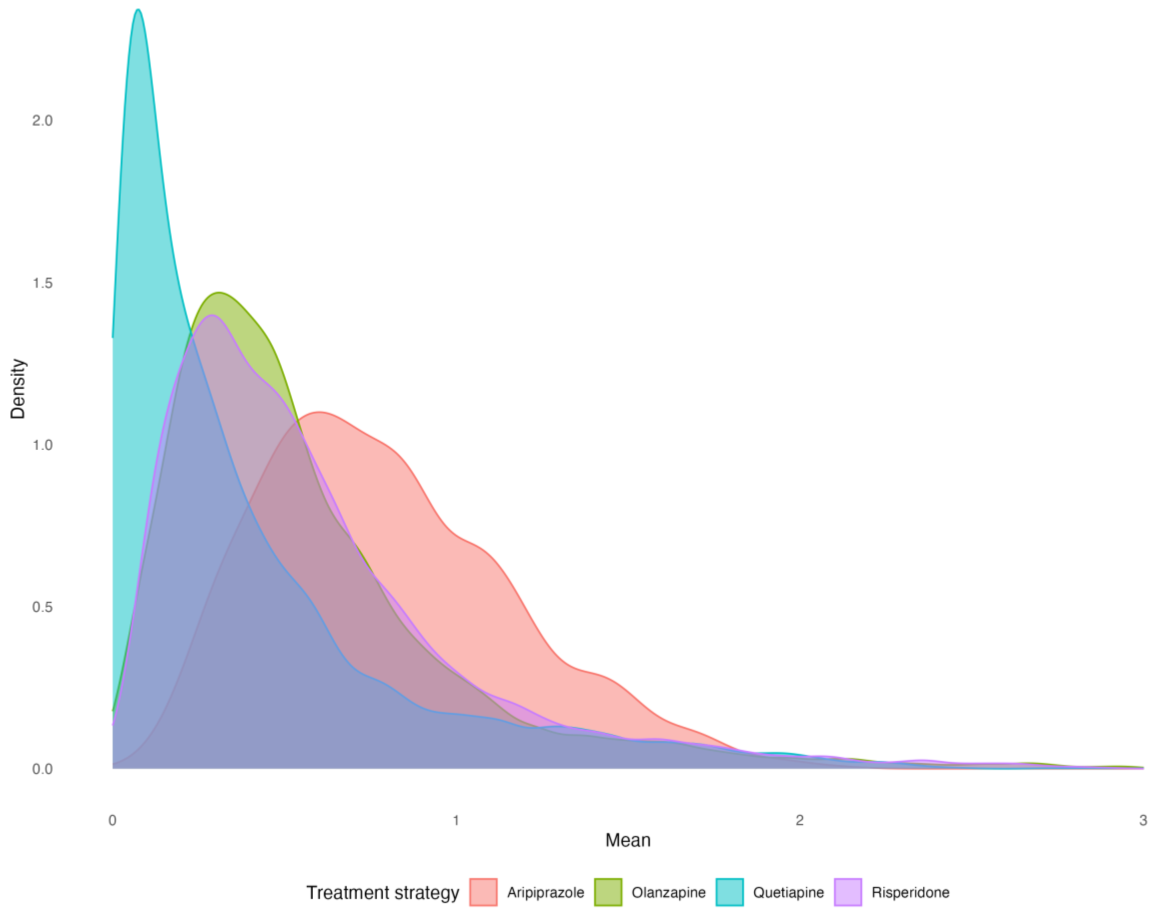

Supplementary Figure 2. Distribution of mean weights for each treatment strategy across imputed datasets.

The raw total number of patients included was 20,404. Weights were scaled to the mean effective sample size (n=11,129).

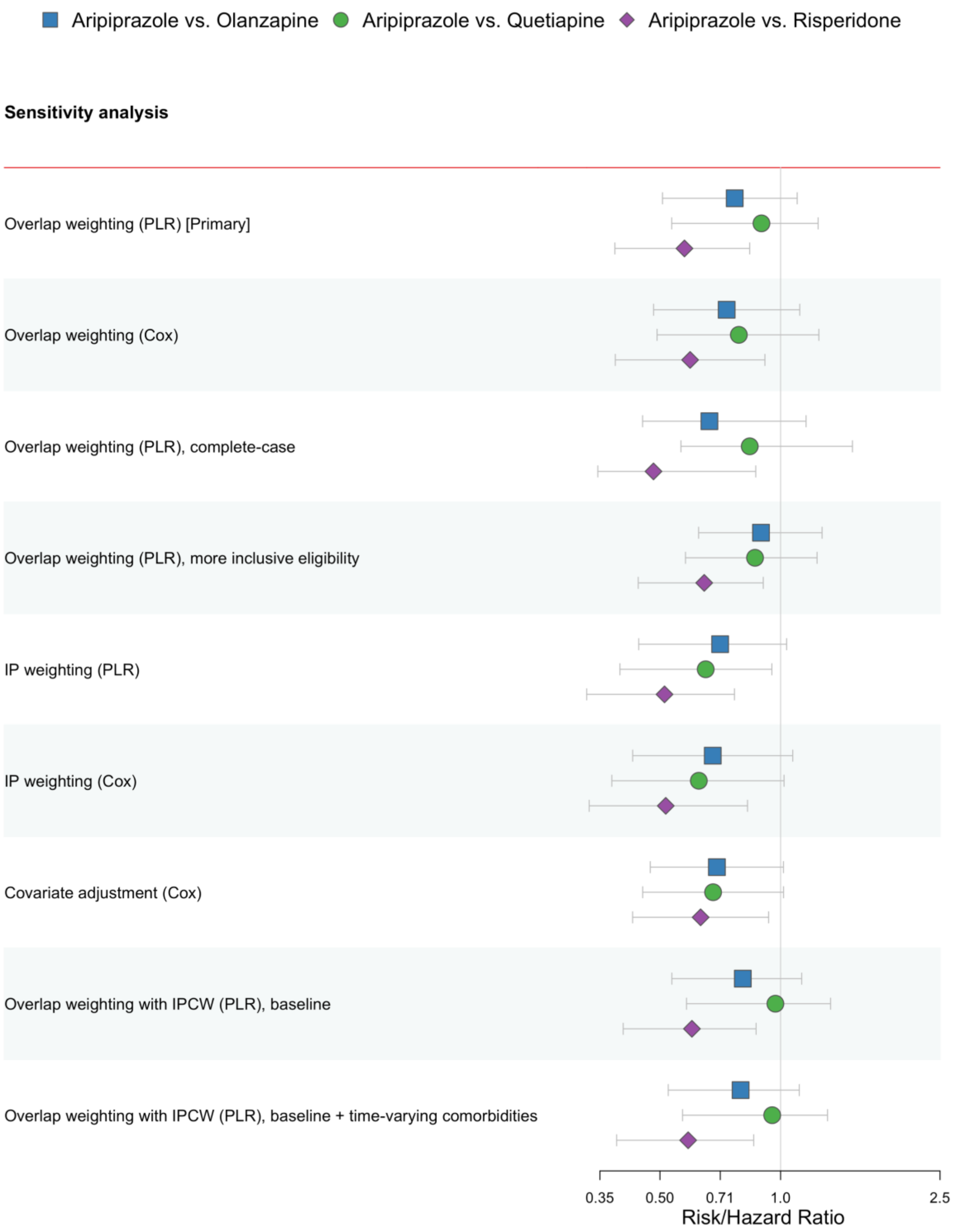

Supplementary Figure 3. Forest plot of per-protocol sensitivity analyses for the five-year risk of MACE.

MACE, major adverse cardiovascular event; PLR, pooled logistic regression; IP, inverse probability; IPCW, inverse probability of censoring weighting.

Estimates are risk or hazard ratios (as appropriate), with 95% confidence intervals (CIs). Risk ratios from the primary analysis (overlap weighting [PLR]) is shown for comparison. CIs for PLR results were derived from the distribution of 2,500 estimates obtained via bootstrapping (i.e., 100 samples per imputed dataset). Bootstrap samples for the IP weighted PLR were increased to 500 due to instability at 100. Source data are provided as a Source Data file.

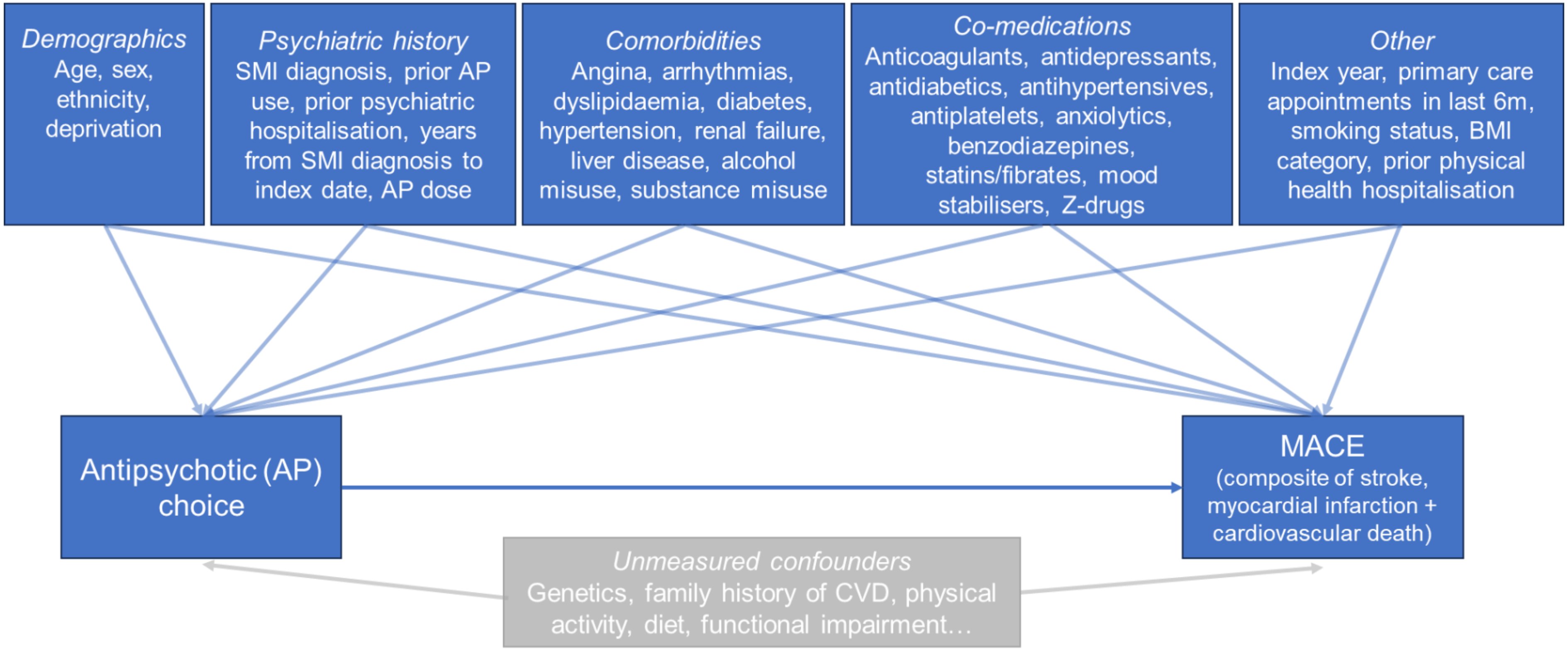

Supplementary Figure 4. Confounding structure directed acyclic graph.

AP, antipsychotic; CVD, cardiovascular disease; MACE, major adverse cardiovascular event; SMI, severe mental illness.

SUPPLEMENTARY REFERENCES

1. Richards-Belle, A. *et al.* Comparative cardiometabolic safety and effectiveness of aripiprazole in people with severe mental illness: A target trial emulation. *PLOS Medicine* **22**, e1004520 (2025).
2. Collett, D. *Modelling Survival Data in Medical Research*. (Chapman and Hall/CRC, New York, 2015). doi:10.1201/b18041.
3. Leucht, S., Samara, M., Heres, S. & Davis, J. M. Dose Equivalents for Antipsychotic Drugs: The DDD Method. *Schizophr Bull* **42**, S90–S94 (2016).
4. Hernán, M. A. & Robins, J. M. Using Big Data to Emulate a Target Trial When a Randomized Trial Is Not Available. *Am J Epidemiol* **183**, 758–764 (2016).
5. Pishgar, F., Greifer, N., Leyrat, C. & Stuart, E. MatchThem:: Matching and Weighting after Multiple Imputation. *The R Journal* **13**, 292–305 (2021).
6. Greifer, N. cobalt: Covariate Balance Tables and Plots. (2024).
